# WRKY transcription factors in legumes

**DOI:** 10.1186/s12870-018-1467-2

**Published:** 2018-10-17

**Authors:** Hui Song, Weihong Sun, Guofeng Yang, Juan Sun

**Affiliations:** Grassland Agri-husbandry Research Center, Qingdao Agricultural University, Qingdao, 266109 China

**Keywords:** Evolutionary rate, Gene feature, Legume, Ortholog, Paralog, Phylogenetic relationship, WRKY

## Abstract

**Background:**

WRKY transcription factors, so named because of the WRKYGQK heptapeptide at the N-terminal end, are widely distributed in plants and play an important role in physiological changes and response to biotic and abiotic stressors. Many previous studies have focused on the evolution of WRKY transcription factors in a given plant; however, little is known about WRKY evolution in legumes. The gene expression pattern of duplicated WRKY transcription factors remains unclear.

**Results:**

We first identified the WRKY proteins in 12 legumes. We found that the WRKYGQK heptapeptide tended to mutate into WRKYGKK. The Q site in WRKYGQK preferentially mutated, while W, K, and Y were conserved. The phylogenetic tree shows that the WRKY proteins in legumes have multiple origins, especially group IIc. For example, WRKY64 from *Lupinus angustifolius* (LaWRKY64) contains three WRKY domains, of which the first two clustered together in the N-terminal WRKY domain of the group I WRKY protein, and the third WRKY domain grouped in the C-terminal WRKY domain of the group I WRKY protein. Orthologous WRKY genes have a faster evolutionary rate and are subject to constrained selective pressure, unlike paralogous WRKY genes. Different gene features were observed between duplicated WRKY genes and singleton WRKY genes. Duplicated *Glycine max* WRKY genes with similar gene features have gene expression divergence.

**Conclusions:**

We analyzed the WRKY number and type in 12 legumes, concluding that the WRKY proteins have multiple origins. A novel WRKY protein, LaWRKY64, was found in *L. angustifolius*. The first two WRKY domains of LaWRKY64 have the same origin. The orthologous and paralogous WRKY proteins have different evolutionary rates. Duplicated WRKY genes have gene expression divergence under normal growth conditions in *G. max*. These results provide insight into understanding WRKY evolution and expression.

**Electronic supplementary material:**

The online version of this article (10.1186/s12870-018-1467-2) contains supplementary material, which is available to authorized users.

## Background

The WRKY gene family comprises a class of important transcription factors involved in physiological change and response to biotic and abiotic stress [1–4]. WRKY transcription factors contain a WRKYGQK heptapeptide at the N-terminal end and a zinc-finger motif (CX_4-5_CX_22-23_HXH or CX_7_CX_23_HXC) at the C-terminal end [1, 2]. WRKY proteins can be classified into groups I–III based on the number of WRKY domains and the type of zinc-finger motif [1, 2]. Group I WRKY contains two WRKY domains and a zinc-finger motif [1, 2]. Group II WRKY contains a single WRKY domain and a CX_4-5_CX_22-23_HXH zinc-finger motif, and group II WRKY can be divided into the following five subgroups: IIa, IIb, IIc, IId, and IIe [1, 2]. Group III WRKY has a single WRKY domain and a CX_7_CX_23_HXC zinc-finger motif [1, 2]. WRKY can also be classified into two groups based on their intron type, R-type or V-type [2]. R-type introns are distributed in groups Ic, IIc, IId, and III, which contains an intron spliced immediately after the R (Arg) position [2, 5, 6]. V-type introns are distributed in groups IIa and IIb. One intron is located before the K (Val) position, which is at the sixth amino acid after the second C residue in the C_2_H_2_ zinc finger motif [2, 5, 6].

WRKY transcription factors can activate downstream genes, involving physiological change and response to biotic and abiotic stress by binding *cis*-acting elements [1, 2]. WRKY transcription factors are involved in seed development [7, 8], seed dormancy and germination [9–11], flowering [12, 13], senescence [14], metabolic pathways [7], morphogenesis of trichomes [15], and plant growth [16]. In addition, WRKY transcription factors, especially group III, can be involved in response to herbivores, pathogens, and nematodes [1, 17–21]. Furthermore, researchers have found that WRKY also aids plant resistance to abiotic stress such as high temperature, low temperature, salt and drought, H_2_O_2_, and UV radiation [22–30].

Many studies have focused on the evolution of the WRKY gene family, but there is a debate about the origin of each type of WRKY in various plant species. According to a phylogenetic tree, the evolutionary relationships proposed revealed that the WRKY gene family can be classified into four clades including groups I + IIc, groups IIa + IIb, group IId, and group IIe [6]. Based upon phylogenetic analyses, researchers have proposed that the group II and III WRKY domains are descendants that have originated from the C-terminal WRKY domain of group I [2, 6]. With the development of sequencing technology, an increasing number of complete genome-wide sequences have been reported for various plant species. Researchers have identified more WRKY gene families in various plants, obtaining results that contrasted the above conclusion. Specifically, Zhu et al. [31] found that the *Triticum aestivum* subgroup IIc WRKY domains originated from the N-terminal WRKY domain of group I. Wei et al. [32] demonstrated that group I WRKY proteins first appeared in monocotyledons, followed by groups III and II. Brand et al. [33] reported that group I and other WRKY proteins likely originated from subgroup IIc. Recently, Rinerson et al. [4] detected the number and type of WRKY gene families ranging from lower organisms to higher organisms without the use of phylogenetic trees. Rinerson et al. [4] proposed two alternative hypotheses of WRKY protein evolution: the “Group I Hypothesis” and the “IIa + b Separate Hypothesis” [4]. The “Group I Hypothesis” proposed that all WRKY proteins evolved from the C-terminal WRKY domains of group I proteins, whereas the “IIa + b Separate Hypothesis” suggested that groups IIa and IIb evolved directly from a single domain algal gene separated from a group I-derived lineage [4].

**Fig. 1:**
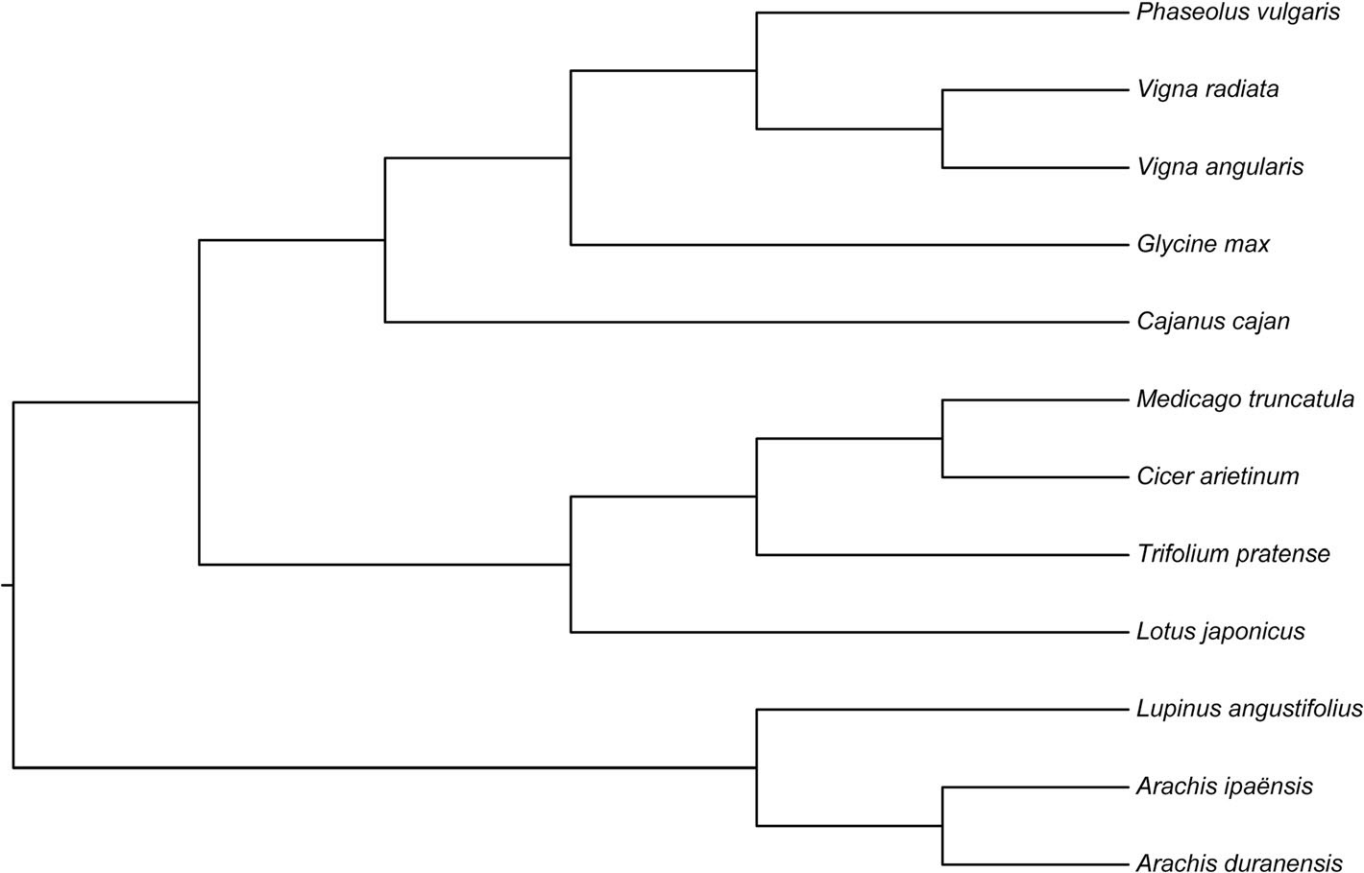
A phylogenetic tree of 12 legume species. The topology of the phylogenetic tree is revised based on references [34–36]

To date, genome-wide sequences have been reported for 12 legume species, and their phylogenetic relationships have been revealed based on genomic data [34–36] (Fig. 1). However, studies on the phylogenetic relationships of the WRKY gene family are limited in these legume species. In this study, we identified WRKY genes using the same method utilized by other studies and confirmed the number and type of WRKY genes in each genome. Then, we identified orthologs and paralogs and estimated their evolutionary rate. Further, we compared gene features between duplicated WRKY genes and singleton WRKY genes as well as the gene features and expression in WRKY paralogs. These results provide a greater depth of the understanding of WRKY evolution.

## Methods

### Identification of WRKY transcription factors in 12 legumes

A total of 12 legumes have reported genome sequences including *Arachis duranensis* (V14167.a1), *Arachis ipaënsis* (K30076.a1), *Cajanus cajan* (Cc 1.0), *Cicer arietinum* (cicar.CDCFrontier.v1.0), *Glycine max* (Wm82.a2), *Lotus japonicus* (Lj3.0), *Lupinus angustifolius* (La1.0), *Medicago truncatula* (Mt4.0), *Phaseolus vulgaris* (V10), *Trifolium pratense* (Tp2.1), *Vigna angularis* (Va3.0), and *Vigna radiata* (Vr1.0) [35–45]*.* These 12 legume genome sequences were downloaded from their sequencing websites (data downloaded on 2017-6-25).

There are many methods currently available to identify gene families in a genome. For example, the similarity-based method [46] and hidden Markov models (HMMs) method. Here, we used the HMMs method to identify the WRKY gene family in the 12 legumes, because this method has high accuracy and sensitivity. Additionally, this method has also been previously used to detect the WRKY gene family in *A. duranensis*, *A. ipaënsis*, and *G. max* [18, 29]. However, the WRKY gene family was identified in *C. arietinum*, *L. japonicus*, and *M. truncatula* using the similarity-based method [47–49]. In this study, we re-identified this gene family in the previously investigated genomes using the HMMs method. In addition, little is known about the WRKY gene family in *C. cajan*, *L. angustifolius*, *P. vulgaris*, *T. pratense*, *V. angularis*, and *V. radiata*. We also used the HMMs method to identify the WRKY gene family in these genomes. In brief, the HMM profile of the WRKY domain (PF03106) was downloaded from the Pfam protein family database (http://pfam.xfam.org/) [50] and was used to survey all proteins using the HMMER program [51]. To verify the reliability of the search results, each protein sequence was checked in the Pfam database. In this study, we excluded the WRKY sequences that had lost the WRKYGQK motif or the zinc finger motif.

### Phylogenetic analyses

The multiple sequence alignment of all WRKY domains and groups I, II, and III domains were executed using MAFFT 7.0 [52]. The maximum likelihood (ML) estimate used the best-fitting model of sequence evolution as determined by ProtTest [53]. The phylogenetic trees were constructed using IQ-TREE [54] and were estimated using an SH-aLRT test with 1000 random addition replicates and ultrafast bootstrap approximation set to 10,000.

For *Arachis*, homologous genes were identified in the transcriptome assembly using the similarity-based method [37, 55]. Here, we used the same method to identify WRKY orthologs and paralogs in the 12 legumes. In brief, the multiple alignment of coding sequences (CDSs) was executed using each species with the local BLAST program [56]. The following evaluation criteria were used as thresholds to determine inclusion in subsequent analyses [55]: (1) length of aligned sequences > 80% of each sequence length; (2) identity > 80%; and (3) E-value ≤10^− 10^. MAFFT 7.0 [52] was used to align duplicated CDS and amino acid pairs. PAL2NAL [57] was used for the conversion of amino acid sequences into the corresponding CDSs. PAML 4.0 [58] was used to calculate the nonsynonymous substitution rates (*K*_*a*_) and synonymous substitution rates (*K*_*s*_). If the *K*_*s*_ value was less than 0.01 or more than 3, and the *K*_*a*_ value was nearly 0, these duplicated genes were excluded, because low sequence divergence could result in unknown estimates, and a high *K*_*s*_ value indicated potential sequence saturation [58, 59]. When *K*_*a*_/*K*_*s*_ = 1 it indicated neutral selection, when it was > 1 it indicated positive selection, and when it was < 1 it indicated purifying selection.

### Gene feature in homologs

To compare duplicated and singleton WRKY genes, we estimated the gene features between these genes. The gene features included polypeptide length, GC content at three codon positions (GC1, GC2, and GC3), and the frequency of optimal codons (Fop). The Fop and polypeptide length were calculated using the codon W program (version 1.4, http://codonw.sourceforge.net). The GC content was estimated using the in-house perl script.

### *Glycine max* transcriptome data under normal growth conditions

The normalized data (Reads/Kb/Million, RPKM) for 14 *G. max* tissues collected during different growth periods, including young leaves, flowers, one cm pods, pod shell 10 days after flowering (DAF), pod shell 14 DAF, seeds 10 DAF, seeds 14 DAF, seeds 21 DAF, seeds 25 DAF, seeds 28 DAF, seeds 35 DAF, seeds 42 DAF, roots, and nodules, was reported by Severin et al. [60]. This data was downloaded from the SoyBase website [61]. The RPKM value was log_2_-transformed as gene expression.

## Results

### WRKY genes in 12 legume species

A total of 75 AdWRKY, 77 AiWRKY, and 178 GmWRKY proteins with both WRKYGQK heptapeptide and zinc-finger motifs were identified in *A. duranensis*, *A. ipaënsis*, and *G. max* genome sequences, respectively [18, 29]. Re-identification of WRKY proteins in *C. arietinum*, *L. japonicus*, and *M. truncatula* showed that 58 CaWRKY, 78 LjWRKY, and 98 MtWRKY proteins, respectively, were found using the HMMs method (Table 1). We detected 92 CcWRKY, 108 LaWRKY, 88 PvWRKY, 89 TpWRKY, 77 VaWRKY, and 76 VrWRKY proteins that contained both the WRKYGQK heptapeptide and zinc-finger motif using the HMMs method in *C. cajan*, *L. angustifolius*, *P. vulgaris*, *T. pratense*, *V. angularis*, and *V. radiata* genomes, respectively (Table 1). In addition, genomes with missing zinc-finger motifs and/or partial WRKY proteins are presented in Additional file 1: Table S1. We named WRKYs based on the order of genes located in chromosomes. CaWRKY, LjWRKY, and MtWRKY have been named in previous studies [47–49]. Newly detected WRKY genes from *C. arietinum*, *L. japonicus*, and *M. truncatula* were named based on the order of their location on the chromosome. If alternative splicing was observed for the WRKY genes, we retained the primary transcript. These WRKY genes can be classified into three groups: groups I, II, and III. A comparison of the number of WRKY genes among the 12 legumes showed that *G. max* contained the greatest number of WRKY genes, while *C. arietinum* contained the fewest WRKY genes.

**Table 1:** The number and type of WRKY proteins in 12 legume species

	Group I	Group II	Group III	Total
*Arachis duranensis*	16	46	13	75
*Arachis ipaënsis*	14	48	15	77
*Cajanus cajan*	16	63	13	92
*Cicer arietinum*	12	39	7	58
*Glycine max*	30	124	24	178
*Lotus japonicus*	14	56	8	78
*Lupinus angustifolius*	24	72	12	108
*Medicago truncatula*	16	64	18	98
*Phaseolus vulgaris*	14	62	12	88
*Trifolium pratense*	14	60	15	89
*Vigna angularis*	15	53	9	77
*Vigna radiata*	16	48	12	76

**Fig. 2:**
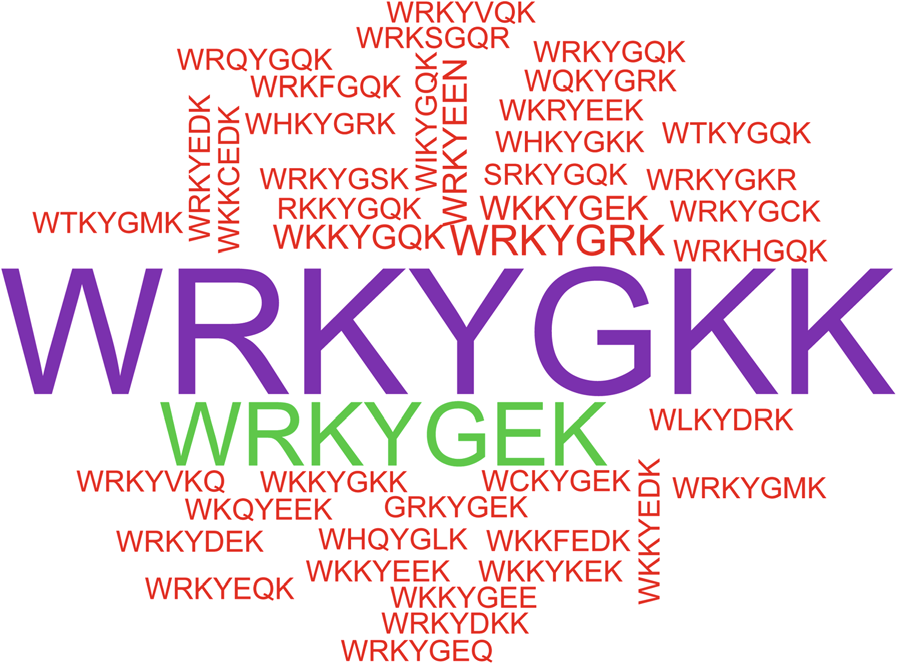
The mutated WRKY domain. The figure was constructed using the wordcloud package in R script. The font size indicates the number of mutations

**Fig. 3:**
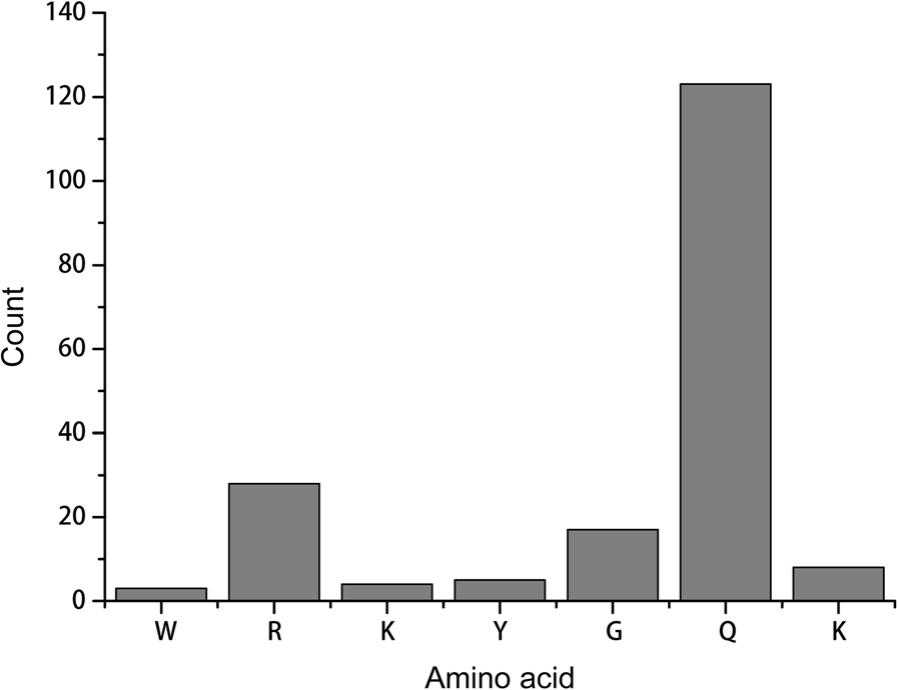
The number of amino acids of the WRKYGQK heptapeptide. The figure was constructed using Origin 9.0. The Y-axis indicates the count, and the X-axis indicates the amino acid

WRKY genes can regulate downstream genes that are involved in physiological change and response to biotic and abiotic stress by WRKYGQK heptapeptide binding to the *cis*-acting element of the downstream gene [1]. In this study, we found that 139 WRKYGQK sequences were observed in at least one mutation site (Fig. 2). WRKYGKK tended to be mutated. Among these sequences, most WRKYGQK sequences from group II WRKY genes appeared to be mutated (Additional file 1: Table S1), indicating that the biological function of the group II WRKY genes was more diverse. Further, in this study, the Q in WRKYGQK preferentially mutated, while the W, K, and Y were conserved (Fig. 3). Previous studies have revealed that mutation of the K positions in WRKYGQK sequences disrupt protein-DNA interactions [62, 63]. In this study, some WRKYGQK sequences were observed with a K mutation, suggesting that the mutation influences protein-DNA interactions.

**Fig. 4:**
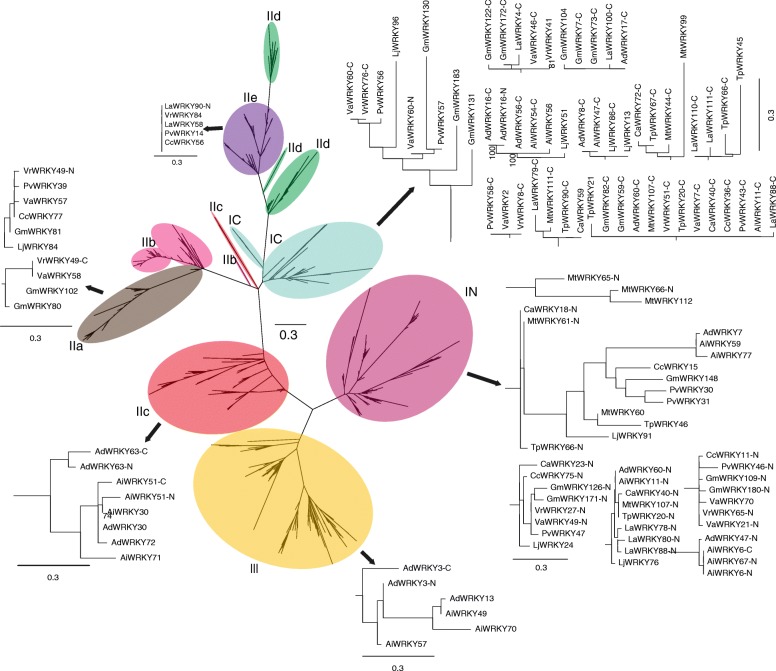
A phylogenetic tree of the WRKY domains in 12 legumes. The phylogenetic tree was constructed using IQ-tree. The phylogenetic tree was estimated using maximum likelihood with the Jones-Taylor-Thornton (JTT) model, and branch support estimates are based on 1000 bootstrap replicates. The legume species included are *Arachis duranensis* (V14167.a1), *Arachis ipaënsis* (K30076.a1), *Cajanus cajan* (Cc 1.0), *Cicer arietinum* (cicar.CDCFrontier.v1.0), *Glycine max* (Wm82.a2), *Lotus japonicus* (Lj3.0), *Lupinus angustifolius* (La1.0), *Medicago truncatula* (Mt4.0), *Phaseolus vulgaris* (V10), *Trifolium pratense* (Tp2.1), *Vigna angularis* (Va3.0), and *Vigna radiata* (Vr1.0)

A WRKY protein can form a fusion protein with the nucleotide binding site-leucine-rich repeat (NBS-LRR) protein [1, 4], which is involved in plant response to pathogens [64]. We found two, one, and one WRKY-NBS fusion proteins in *A. duranensis*, *A. ipaënsis*, and *G. max*, respectively. We propose that there is no evolutionary relationship among WRKY proteins, NBS-LRR proteins, and WRKY-NBS fusion proteins due to the absence of WRKY-NBS proteins in close relatives to *A. duranensis*, *A. ipaënsis*, and *G. max*. WRKY-NBS fusion proteins can be classified into eight groups based on a phylogenetic tree [4]. As reported in a previous study, a *G. max* WRKY-NBS protein that belongs to the group RW4 was identified [4], but there is no record of the *Arachis* WRKY-NBS fusion protein. We constructed an ML phylogenetic tree using the JTT + I + G model using reported WRKY-NBS fusion proteins and *Arachis* WRKY-NBS fusion proteins. The phylogenetic tree showed that *Arachis* WRKY-NBS fusion proteins are a part of the group RW4 (Additional file 2: Figure S1). In addition, an ML phylogenetic tree using the JTT + G model based upon 12 legume WRKY domains was constructed (Fig. 4 and Additional file 3: Figure S2). GmWRKY, AdWRKY, AiWRKY, MtWRKY, LjWRKY, and CaWRKY have been reported in previous studies [18, 29, 47–49]. Accordingly, we used these WRKY genes as reference sequences to further classify other group II WRKY genes. The ML tree showed that the WRKY proteins can be classified into eight clusters: In, Ic, IIa, IIb, IIc, IId, IIe, and III (Fig. 4). However, we found that some clades contain different types of WRKY proteins. For example, some group II WRKY proteins clustered with group I WRKY proteins, and some group I WRKY proteins mixed with groups IIa, IIc, IIe, and III (Fig. 4). Further, two WRKY domains of group I grouped with IIa, IIc, and III, but only N-terminal WRKY domains of group I clustered in group IIe. In addition, the WRKY proteins in groups In, IIa, IIe, and III clustered into separate clades, but the other types of WRKY proteins formed multiple mixed clades (Fig. 4). These results indicated that WRKY proteins have multiple origins, especially group IIc.

**Fig. 5:**
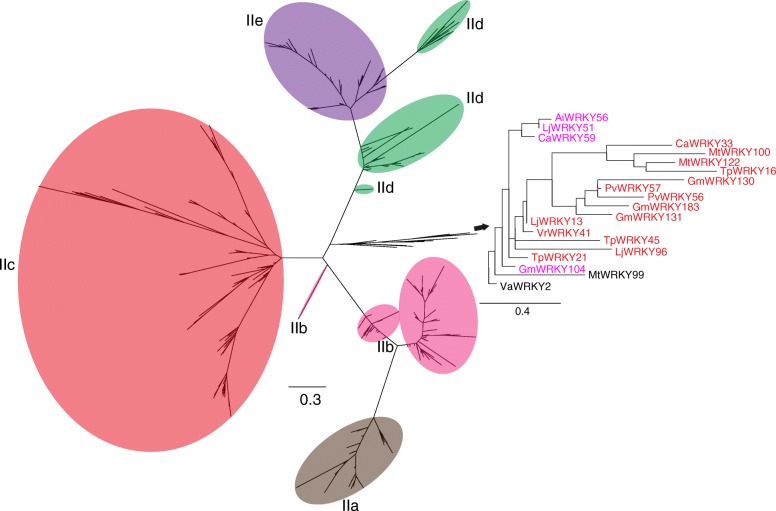
A phylogenetic tree of the WRKY group II domain in 12 legumes. The phylogenetic tree was constructed using IQ-tree. The phylogenetic tree was estimated using maximum likelihood with the Jones-Taylor-Thornton (JTT) model, and branch support estimates are based on 1000 bootstrap replicates. The legumes included are *Arachis duranensis* (V14167.a1), *Arachis ipaënsis* (K30076.a1), *Cajanus cajan* (Cc 1.0), *Cicer arietinum* (cicar.CDCFrontier.v1.0), *Glycine max* (Wm82.a2), *Lotus japonicus* (Lj3.0), *Lupinus angustifolius* (La1.0), *Medicago truncatula* (Mt4.0), *Phaseolus vulgaris* (V10), *Trifolium pratense* (Tp2.1), *Vigna angularis* (Va3.0), and *Vigna radiata* (Vr1.0)

To reveal the phylogenetic relationships of group II WRKY proteins, we constructed an ML phylogenetic tree using the JTT + G model based on all group II WRKY proteins in the 12 legumes (Fig. 5 and Additional file 4: Figure S3). The topological structure of the phylogenetic tree is similar between using solely group II WRKY proteins and using all WRKY proteins. For example, groups IIa and IIe clustered in a clade, and other WRKY proteins grouped into multiple clades. However, compared with using all WRKY proteins to construct the phylogenetic tree, the number of clusters of group IIc WRKY proteins is relatively lower. We found a mixed clade, IIm, including group IIb and IIc WRKY proteins. Further, we found that members of the clade IIm were observed in group II, including MtWRKY99 and VaWRKY2, but were not classified (Fig. 5). These results indicated that the IIm WRKY proteins have multiple origins.

**Fig. 6:**
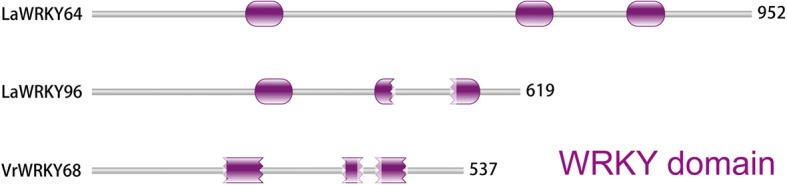
The WRKY domains in three WRKY proteins. The three amino acids are uploaded on the Pfam database (http://pfam.xfam.org/), and the domain structure was constructed in Pfam

The number of WRKY domains showed that LaWRKY64 contains three WRKY domains (Fig. 6), and we propose this newly identified sequence as a member of group I. In addition, LaWRKY96 and VrWRKY68 contain three WRKY domains, but the second and third WRKY domain of LaWRKY96 and VrWRKY68 are abnormal. We speculate that the original LaWRKY96 and VrWRKY68 contain two WRKY domains because there is a short sequence inserted into the second WRKY domain in LaWRKY96 and VrWRKY68 (Fig. 6). The phylogenetic tree showed that the first two WRKY domains of LaWRKY64 clustered together in In, and the third WRKY domain grouped in Ic (Fig. 4). Accordingly, we proposed that the second WRKY domain possibly originated from the first WRKY domain in LaWRKY64. To verify this hypothesis, we constructed a phylogenetic tree using group I WRKY proteins. The results showed that the sequences were highly homologous between the first and second WRKY domains (Additional file 5: Figure S4).

### Evolutionary rate and gene feature in homologous genes

**Fig. 7:**
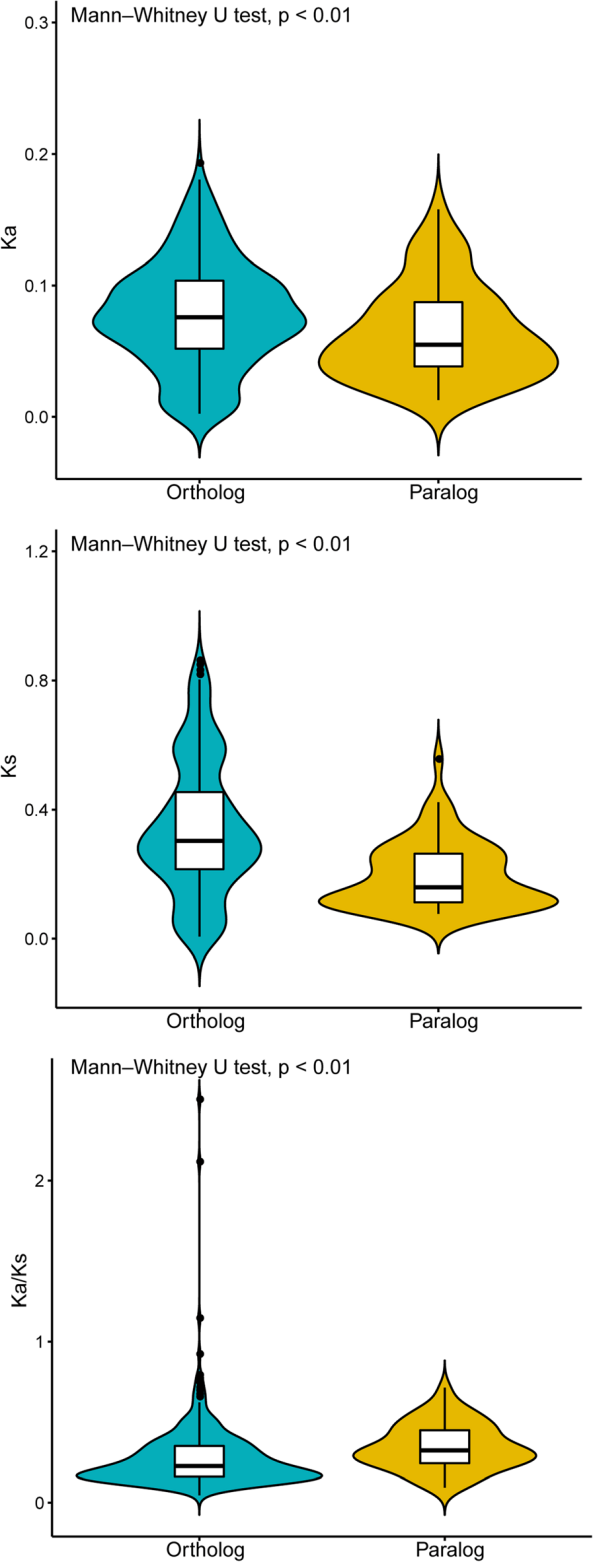
Comparison of the evolutionary rate between orthologs and paralogs. PAL2NAL was used to convert amino acid sequences into the corresponding nucleotide sequences. PAML 4.0 was used to calculate the nonsynonymous/synonymous substitution (*K*_a_/*K*_s_) rate. The figure was constructed using the ggpubr package in R script

In this study, we identified 317 orthologous pairs and 67 paralogous pairs in 12 legumes. We excluded one orthologous pair and six paralogous pairs due to the abnormality of the evolutionary rates of these seven homologous pairs. The evolutionary rate showed that the average *K*_*a*_, *K*_*s*_, and *K*_*a*_/*K*_*s*_ of the paralogs was 0.06, 0.19, and 0.34, respectively. The average *K*_*a*_, *K*_*s*_, and *K*_*a*_/*K*_*s*_ of the orthologs was 0.08, 0.34, and 0.28, respectively. A comparison of paralogs and orthologs revealed that the average *K*_*a*_ and *K*_*s*_ (a proxy for evolutionary rate) of the orthologs was higher than that of the paralogs, but the *K*_*a*_/*K*_*s*_ (a proxy for selective pressure) for the orthologs was lower than that of the paralogs (Fig. 7). These results indicated that orthologs have a faster evolutionary rate and are subject to constrained selective pressure, unlike paralogs.

The correlation analyses showed that *K*_*a*_ was positively correlated with *K*_*s*_ in paralogs and orthologs (paralog: *r* = 0.73, *P* < 0.01; ortholog: *r* = 0.58, *P* < 0.01). The *K*_*s*_ was negatively correlated with the *K*_*a*_/*K*_*s*_ in paralogs and orthologs (paralog: *r* = − 0.27, *P* < 0.05; ortholog: *r* = − 0.42, *P* < 0.01). The *K*_*a*_ was not correlated with the *K*_*a*_/*K*_*s*_ in orthologs (ortholog: *r* = 0.07, *P* > 0.05), but the *K*_*a*_ was significantly positively correlated with the *K*_*a*_/*K*_*s*_ in paralogs (paralog: *r* = 0.38, *P* < 0.01). These results indicated that *K*_*a*_ influenced the *K*_*a*_/*K*_*s*_ in paralogs, while the *K*_*s*_ determined the *K*_*a*_/*K*_*s*_ in paralogs and orthologs.

Comparison of gene features between paralogs and orthologs showed that the average Fop and GC content of the orthologs was not significantly lower than that of paralogs (Additional file 6: Table S2).

### Correlation of evolutionary rate and gene feature between orthologs and paralogs

There are correlations between evolutionary rate and gene feature [59, 65, 66]; however, different correlations have been found in various organisms. In this study, our results showed that the *K*_*a*_ was significantly negatively correlated with Fop, GC1, and GC2, and the *K*_*a*_/*K*_*s*_ was significantly negatively correlated with Fop, polypeptide length, GC1, and GC2 in orthologs (Table 2). In paralogs, the *K*_*a*_ was significantly negatively correlated with Fop, GC2, and GC3, and the *K*_*a*_/*K*_*s*_ was significantly negatively correlated with Fop, GC1, GC2, and GC3 (Table 2). These results indicated that both Fop and GC2 influenced in *K*_*a*_, and Fop, GC1, and GC2 affected *K*_*a*_/*K*_*s*_ in paralogs and orthologs.

**Table 2:** The correlation of evolutionary rate and gene feature in WRKY orthologs and paralogs

	Fop	Polypeptide length	GC1	GC2	GC3
Ortholog					
*K*_*s*_	0.09026	−0.0958	− 0.10494	− 0.0116	0.0672
*K*_*a*_	− 0.18988**	− 0.10997	− 0.25635**	− 0.25051**	− 0.06667
*K*_*a*_*/K*_*s*_	− 0.18418**	− 0.15026**	−0.23272**	− 0.23049**	−0.0526
Paralog					
*K*_*s*_	0.10751	0.09914	−0.08703	0.03401	−0.10284
*K*_*a*_	−0.36815**	0.11903	−0.24856	−0.36392**	− 0.37883**
*K*_*a*_*/K*_*s*_	−0.58738**	− 0.04473	−0.2972*	− 0.54631**	−0.29905*

*Fop* frequency of optimal codons, *GC1* GC content at first codon positions, *GC2* GC content at second codon positions, *GC3* GC content at third codon positions, *K*_*s*_ synonymous substitution ratio, *K*_a_ nonsynonymous substitution ratio, *K*_a_/*K*_s_ nonsynonymous to synonymous substitution ratio

*indicates significance at *P* < 0.05; **indicates significance at *P* < 0.01

### Comparison of gene features between duplicated and singleton WRKY genes

Here, we addressed the gene features between duplicated WRKY genes and singleton WRKY genes in legumes. We found that the Fop (a proxy for codon usage bias) of duplicated WRKY genes was slightly higher than that of singleton WRKY genes (Table 3). The polypeptide length of duplicated WRKY genes was longer than that of singleton WRKY genes (Table 3). The GC content at the three codon positions of duplicated WRKY genes was higher than that of singleton WRKY genes (Table 3), but this is not statistically significant in GC1 content (Table 3). These results indicated that different gene features were observed between duplicated WRKY genes and singleton WRKY genes.

**Table 3:** Comparison of gene features between duplicate and singleton WRKY genes

	^a^Duplicate	^a^Singleton	*P* value
Fop	0.3999 ± 0.0344	0.3910 ± 0.0443	0.014
Polypeptide length	398 ± 144	376 ± 164	0.014
GC1	47.5282 ± 4.5553	46.6569 ± 4.3516	0.0981
GC2	43.5792 ± 4.2996	42.1790 ± 4.5924	0.0189
GC3	42.3161 ± 7.7402	37.2784 ± 7.6023	1.49E-10

*Fop* frequency of optimal codons, *GC1* GC content at first codon positions, *GC2* GC content at second codon positions, *GC3* GC content at third codon positions

^a^Mean ± SD

### *Glycine max* WRKY paralogs

Compared with WRKY genes in other legumes, more duplicated GmWRKY (41 gene pairs) and LaWRKY (19 gene pairs) genes remained during the evolutionary process. Therefore, we investigated the gene features, evolutionary rates, and gene expression patterns in duplicated WRKY genes. To that end, we used duplicated GmWRKY because multiple tissue transcriptome datasets have been published for *G. max*, and it has the greatest number of duplicated WRKY genes. Our results showed that there is a slight difference in Fop (0–0.073), polypeptide length (0–14), GC1 (0–2.466), GC2 (0–3.087), and GC3 (0.015–4.664) among duplicated GmWRKY genes (Additional file 7: Table S3). However, the gene expression levels of 14 different tissues were observed to be largely different in duplicated GmWRKY genes. The differential gene expression of greater than 50% of the duplicated WRKY genes was observed as up to a two-fold difference, except for duplicated GmWRKY genes in seed tissue (Additional file 7: Table S3). These results indicated that the duplicated WRKY genes with similar gene features have gene expression divergence.

Negative correlations were found between the *K*_*a*_/*K*_*s*_ and the gene expression level in 14 different tissues, but no statistical significance was found in young leaves, flowers, pod shells, and nodule tissues (Table 4). The *K*_*a*_ was significantly negatively correlated with the gene expression level in seeds 14 DAF and root tissues (Table 4). However, the *K*_*s*_ had a coefficient of irregularity with the gene expression level, but it was not statistically significant (Table 4). The correlation between gene features and gene expression level revealed that gene expression level was positively correlated with Fop, GC2, and GC3, but was not statistically significantly different in some tissues (Table 4). For example, the gene expression levels of leaves and pods 14 DAF were positively correlated with Fop, but were not statistically significantly different. There is correlation but no significance between the gene expression level of pods and GC2 (Table 4). In addition, the gene expression level was significantly positively correlated with GC3 in seeds 14 DAF, roots, and nodule tissues (Table 4). The correlations among gene expression level, polypeptide length, and GC1 were irregular and not statistically significant (Table 4).

**Table 4:** The correlation between evolutionary rate, gene feature, and gene expression in *Glycine max* duplicates

	Young leaf	Flower	One cm pod	Pod shell 10DAF	Pod shell 14DAF	Seed 10DAF	Seed 14DAF	Seed 21DAF	Seed 25DAF	Seed 28DAF	Seed 35DAF	Seed 42DAF	Root	Nodule
*K*_*s*_	0.16331	0.0698	−0.0229	0.10568	0.10625	−0.06594	0.1175	0.19648	0.18418	0.15055	0.21299	0.2218	−0.07009	0.03759
*K*_*a*_	−0.06796	−0.1692	− 0.29357	−0.14733	− 0.08252	−0.3319*	− 0.23597	−0.11462	− 0.21668	−0.20247	− 0.09324	−0.1118	− 0.4617**	−0.1818
*K*_*a*_ */K*_*s*_	−0.20271	−0.2411	− 0.31353*	−0.27015	− 0.12681	−0.36376*	− 0.42364**	−0.32785*	− 0.46039**	−0.42072**	− 0.36024*	−0.4012**	− 0.52122**	−0.23178
Fop	0.29568	0.30819*	0.34913*	0.35181*	0.26262	0.38268*	0.39778*	0.3624*	0.49315**	0.53284**	0.44177**	0.50804**	0.36783*	0.43526**
Polypeptide length	0.06744	0.04395	0.08372	−0.00281	−0.07692	0.16338	0.12091	0.1345	0.18838	0.20758	0.22477	0.19481	0.09388	0.20409
GC1	0.01633	0.05774	0.3434*	0.00975	−0.14317	0.38559*	0.32063*	0.32252*	0.23863	0.2538	0.21561	0.22572	0.40488**	0.22962
GC2	0.32679*	0.32974*	0.42521**	0.29401	0.21021	0.50622**	0.52072**	0.46359**	0.57195**	0.57169**	0.48728**	0.47168**	0.65552**	0.46916**
GC3	0.16546	0.20181	0.28002	0.16329	0.17571	0.25886	0.31821*	0.30376	0.28938	0.28818	0.19329	0.21183	0.45942**	0.31169*

*DAF* day after flowering, *Fop* frequency of optimal codons, *GC1* GC content at first codon positions, *GC2* GC content at second codon positions, *GC3* GC content at third codon positions, *K*_s_ synonymous substitution ratio, *K*_a_ nonsynonymous substitution ratio, *K*_a_/*K*_s_ nonsynonymous to synonymous substitution ratio

*indicates significance at *P* < 0.05; **indicates significance at *P* < 0.01

## Discussion

### WRKY number, structure, and evolution in legumes

To date, genome-wide sequences obtained via different pipelines have been published for 12 legume species [35–45]. Accordingly, the sequencing depth of these legumes varies, making it difficult to compare the number of WRKY genes. However, in this study, the largest number of GmWRKY genes was detected for *G. max* compared with the 11 other legume species. This is because *G. max* is an autotetraploid [34], and more than three whole-genome duplication (WGD) events have been identified for *G. max* [67]. Our previous study revealed that the number of WRKY genes was not correlated with genome size but positively correlated with the number of WGD events [18]. This is also consistent with our other study, in which we found that the number of GmLOX genes is greater than in other legumes [68]. Unexpectedly, *L. angustifolius*, a diploid species, has more WRKY genes than other diploid legumes in this study. One reasonable explanation is the retention rate of duplicated WRKY genes is higher than in other legumes.

Normally, WRKY proteins contain one or two WRKY domains [1, 2]. However, Mohanta et al. [69] found that WRKY proteins contained three WRKY domains in *Gossypium raimondii* and *Linum usitatissimum* and four WRKY domains in *Aquilegia coerulea* and *Setaria italic*. We speculate that WRKY proteins containing three or four WRKY domains might have recently evolved because these types of WRKY proteins have only been observed in higher plant species, not in lower plant species [69]. We also identified a WRKY protein containing three domains in *L. angustifolius*. Our results indicated that the middle WRKY domain possibly produced the N-terminal WRKY domain, indicating that WRKY domains can be replicated using gene duplication.

Genomic rearrangement plays a pivotal force in forming WRKY-NBS fusion proteins [4], and NBS-LRR is a resistance gene involved in response to pathogens [64]. WRKY genes located in central positions mediating fast and efficient activation of defense programs [21]. Accordingly, we speculated that the fusion of WRKY proteins and NBS-LRR proteins can increase disease resistance in plants. The effector PopP2 and AvrRps4 can bind to the WRKY domain of the Arabidopsis WRKY-NBS fusion protein, which activates another NBS-LRR protein involved in response to bacterial pathogens in *Nicotiana benthamiana* and *Nicotiana tabacum* [70].

It is hard to explain the origin of the WRKY gene family using only one hypothesis, such as the “Group I Hypothesis” and “IIa + b Separate Hypothesis” [4]. This is because an increasing number of studies has revealed that the WRKY gene family has multiple origins. The “Group I Hypothesis” proposed that all WRKY proteins evolved from C-terminal WRKY domains of group I proteins, whereas the “IIa + b Separate Hypothesis” stated that groups IIa and IIb evolved directly from a single domain algal gene separated from the group I-derived lineage [4]. Our results are consistence with these two hypotheses. Based on our phylogenetic analyses, we found that WRKY genes from different groups clustered into a clade. Furthermore, our study fills a gap in the knowledge on the evolution of WRKY genes by comparing gene features and evolutionary rates between WRKY orthologs and paralogs. We concluded that (1) *K*_*a*_ influenced *K*_*a*_*/K*_*s*_ in paralogs, while the *K*_*s*_ determined the *K*_*a*_/*K*_*s*_ in paralogs and orthologs; (2) orthologs have a faster evolutionary rate and are subject to constrained selective pressure, unlike paralogs; and (3) both Fop and GC2 influenced *K*_*a*_, and Fop, GC1, and GC2 affected *K*_*a*_*/K*_*s*_ in paralogs and orthologs. However, more studies are required on legume WRKY genes because our current analysis does not clarify the evolutionary relationship among each group of WRKY genes. Therefore, further studies should not only focus on producing phylogenetic trees, but also work towards identifying novel WRKY genes, with a particular focus on WRKY proteins containing more than two domains or WRKY fusion proteins.

### Gene expression in duplicated GmWRKY genes

The copy of duplicated genes will often be lost after WGD or small-scale duplication (SSD) [67]. However, many copies will be retained in the genome because they have novel molecular, structural, or adaptive traits functions [71]. Some researchers have proposed that duplicated genes with the same biological function will be lost due to fitness cost [72, 73]. Others researchers hold that duplication allows further adaptive changes to accumulate [71, 74, 75]. In addition to these contrasting proposals, four mechanisms can explain the retention of duplicates: gene dosage increase [72]; duplication, degeneration, and complementation (DDC) [76]; gene balance [77]; and paralog interference [74]. In this study, we found that the gene features of duplicated gene were similar, but the gene expression patterns of duplicated genes were different in 14 different tissues. The duplicated GmWRKY genes might have been retained because copies had the asymmetric expression pattern when following the explanation of the DDC mechanism. The DDC model proposed that the mutations that cause subfunctionalization are explicitly neutral [67, 76].

In this study, we found a negative correlation between *K*_*a*_*/K*_*s*_ and gene expression level in 14 different tissues from *G. max*. This result is consistence with the expression-rate of sequence evolution anticorrelation model (E-R anticorrelation) [78]. The model proposed that the most highly expressed genes are also subject to the strongest selective constraint [79]. This can be explained by the expression cost hypothesis, the protein misfolding avoidance hypothesis, the protein misinteraction avoidance hypothesis, and the mRNA folding requirement hypothesis [80]. In addition, our results showed that Fop was positively correlated with gene expression levels, indicating that highly expressed genes have high codon usage bias. This is supported by natural selection in highly expressed genes preferentially using optimal codons [81]. Accordingly, we propose that natural selection plays a crucial role in codon usage bias of GmWRKY genes.

## Conclusions

We identified the WRKY proteins in 12 different legume species, then we compared the gene number and type of WRKY proteins among the legumes. We found a novel WRKY protein, LaWRKY64, which contains three WRKY domains. The phylogenetic tree showed that the WRKY proteins in the 12 legumes have multiple origins. Duplicated and singleton WRKY genes have different gene features. Duplicated GmWRKY genes with similar gene features have gene expression divergence.

## Additional files

Additional file 1: **Table S1.** The name, chromosomal location, number, and type of WRKY in 12 legumes. ^a^Null indicates that the protein lacks WRKY features. ^b^The bold font indicates an amino acid mutation. (XLSX 107 kb)

Additional file 2: **Figure S1.** A phylogenetic tree of WRKY-NBS proteins. The phylogenetic tree was constructed using IQ-tree. The phylogenetic tree was estimated using maximum likelihood with the Jones-Taylor-Thornton (JTT) model, and branch support estimates are based on 1000 bootstrap replicates. (TIF 386 kb)

Additional file 3: **Figure S2.** A phylogenetic tree of the WRKY domains in 12 legumes. The phylogenetic tree was constructed using IQ-tree. The phylogenetic tree was estimated using maximum likelihood with the Jones-Taylor-Thornton (JTT) model, and branch support estimates are based on 1000 bootstrap replicates. The legumes included are *Arachis duranensis* (V14167.a1), *Arachis ipaënsis* (K30076.a1), *Cajanus cajan* (Cc 1.0), *Cicer arietinum* (cicar.CDCFrontier.v1.0), *Glycine max* (Wm82.a2), *Lotus japonicus* (Lj3.0), *Lupinus angustifolius* (La1.0), *Medicago truncatula* (Mt4.0), *Phaseolus vulgaris* (V10), *Trifolium pratense* (Tp2.1), *Vigna angularis* (Va3.0), and *Vigna radiata* (Vr1.0). (PDF 1560 kb)

Additional file 4: **Figure S3.** A phylogenetic tree of the WRKY group II domain in 12 legumes. The phylogenetic tree was constructed using IQ-tree. The phylogenetic tree was estimated using maximum likelihood with the Jones-Taylor-Thornton (JTT) model, and branch support estimates are based on 1000 bootstrap replicates. The legumes included are *Arachis duranensis* (V14167.a1), *Arachis ipaënsis* (K30076.a1), *Cajanus cajan* (Cc 1.0), *Cicer arietinum* (cicar.CDCFrontier.v1.0), *Glycine max* (Wm82.a2), *Lotus japonicus* (Lj3.0), *Lupinus angustifolius* (La1.0), *Medicago truncatula* (Mt4.0), *Phaseolus vulgaris* (V10), *Trifolium pratense* (Tp2.1), *Vigna angularis* (Va3.0), and *Vigna radiata* (Vr1.0). (PDF 990 kb)

Additional file 5: **Figure S4.** A phylogenetic tree of the WRKY group I domain in 12 legumes. The phylogenetic tree was constructed using IQ-tree. The phylogenetic tree was estimated using maximum likelihood with the Jones-Taylor-Thornton (JTT) model, and branch support estimates are based on 1000 bootstrap replicates. The legumes included are *Arachis duranensis* (V14167.a1), *Arachis ipaënsis* (K30076.a1), *Cajanus cajan* (Cc 1.0), *Cicer arietinum* (cicar.CDCFrontier.v1.0), *Glycine max* (Wm82.a2), *Lotus japonicus* (Lj3.0), *Lupinus angustifolius* (La1.0), *Medicago truncatula* (Mt4.0), *Phaseolus vulgaris* (V10), *Trifolium pratense* (Tp2.1), *Vigna angularis* (Va3.0), and *Vigna radiata* (Vr1.0). The bold font indicates WRKY proteins with three WRKY domains. (TIF 5840 kb)

Additional file 6: **Table S2.** Comparison of gene features between WRKY orthologs and paralogs. Fop: frequency of optimal codons; GC1: GC content at first codon positions; GC2: GC content at second codon positions; GC3: GC content at third codon positions. ^a^Mean ± SD (XLSX 10 kb)

Additional file 7: **Table S3.** Absolute value of gene feature and gene expression between *Glycine max* WRKY duplicates. DAF: day after flowering; Fop: frequency of optimal codons; GC1: GC content at first codon positions; GC2: GC content at second codon positions; GC3: GC content at third codon positions. (XLSX 16 kb)

## Abbreviations

CDS: Coding sequence
DAF: Day after flowering
DDC: Duplication, degeneration, and complementation
E-R anticorrelation: Expression-rate of sequence evolution anticorrelation model
Fop: Frequency of optimal codons
GC1: GC content at first codon positions
GC2: GC content at second codon positions
GC3: GC content at third codon positions
HMM: Hidden Markov model
*K*_a_: Nonsynonymous substitution ratio
*K*_a_/*K*_s_: Nonsynonymous to synonymous substitution ratio
*K*_s_: Synonymous substitution ratio
ML: Maximum likelihood
NBS–LRR: Nucleotide binding site-leucine-rich repeat
RPKM: Reads/Kb/Million
SSD: Small-scale duplication
WGD: Whole-genome duplication
