# Supplementary figures and images for: WRKY transcription factors in legumes
Source: BMC Plant Biol. 2018 Oct 17;18:243. doi: 10.1186/s12870-018-1467-2 (PMC6192229; doi:10.1186/s12870-018-1467-2)

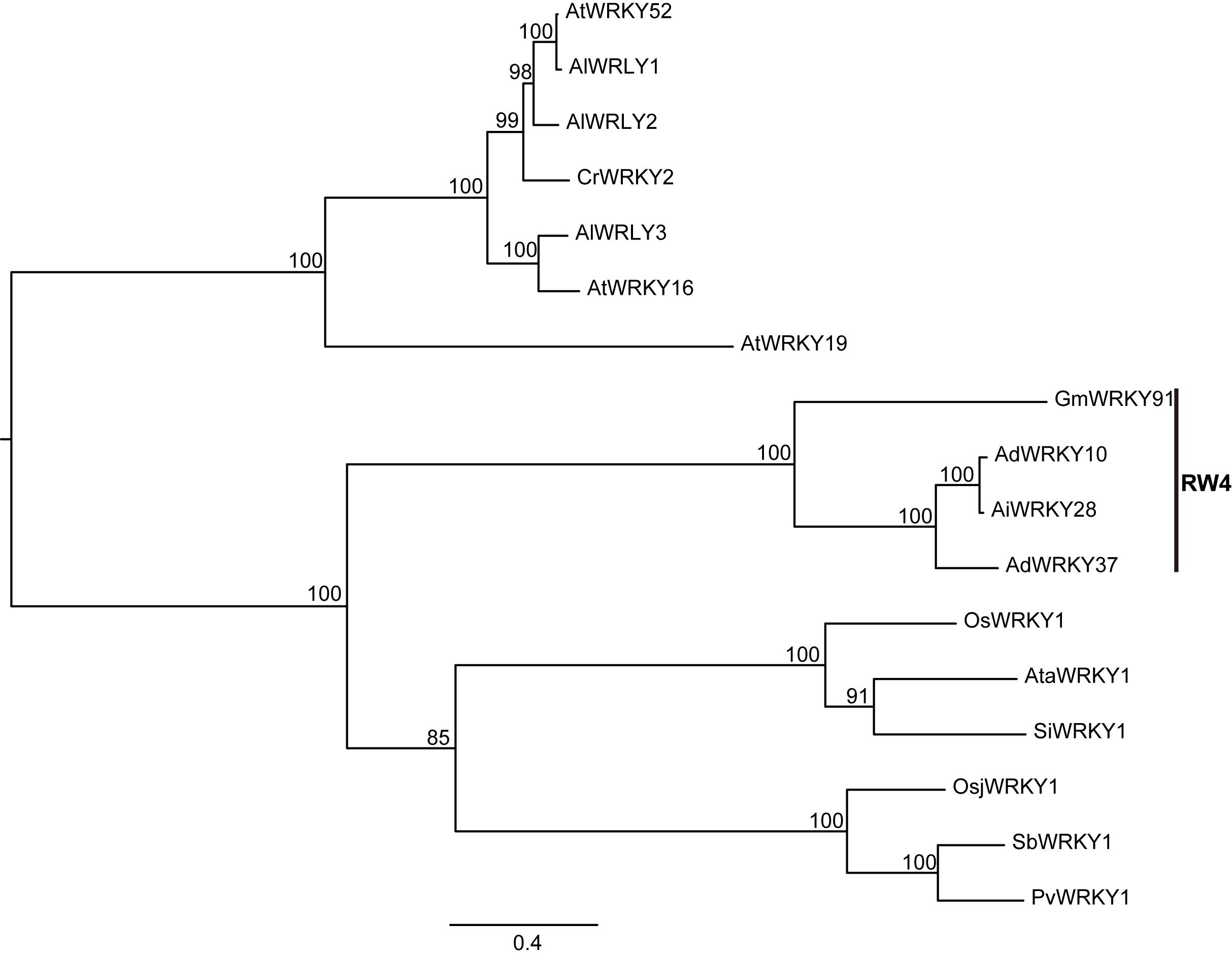

Supplement: Supplementary file 2 — Figure S1. A phylogenetic tree of WRKY-NBS proteins. The phylogenetic tree was constructed using IQ-tree. The phylogenetic tree was estimated using maximum likelihood with the Jones-Taylor-Thornton (JTT) model, and branch support estimates are based on 1000 bootstrap replicates. (TIF 386 kb) [file 12870_2018_1467_MOESM2_ESM.tif]

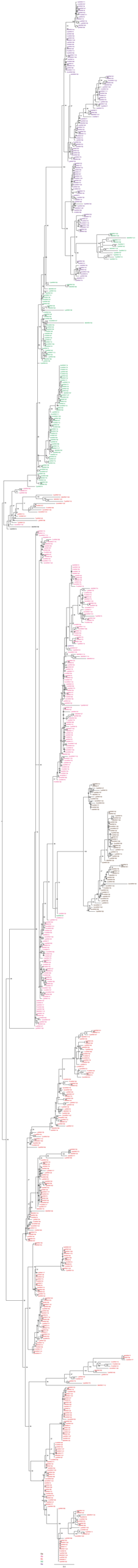

Supplement: Supplementary file 4 — Figure S3. A phylogenetic tree of the WRKY group II domain in 12 legumes. The phylogenetic tree was constructed using IQ-tree. The phylogenetic tree was estimated using maximum likelihood with the Jones-Taylor-Thornton (JTT) model, and branch support estimates are based on 1000 bootstrap replicates. The legumes included are Arachis duranensis (V14167.a1), Arachis ipaënsis (K30076.a1), Cajanus cajan (Cc 1.0), Cicer arietinum (cicar.CDCFrontier.v1.0), Glycine max (Wm82.a2), Lotus japonicus (Lj3.0), Lupinus angustifolius (La1.0), Medicago truncatula (Mt4.0), Phaseolus vulgaris (V10), Trifolium pratense (Tp2.1), Vigna angularis (Va3.0), and Vigna radiata (Vr1.0). (PDF 990 kb) [file 12870_2018_1467_MOESM4_ESM.pdf]
