# Supplementary material for: WRKY transcription factors in legumes
Source: BMC Plant Biol. 2018 Oct 17;18:243. doi: 10.1186/s12870-018-1467-2 (PMC6192229; doi:10.1186/s12870-018-1467-2)
Supplement: Supplementary file 3 — Figure S2. A phylogenetic tree of the WRKY domains in 12 legumes. The phylogenetic tree was constructed using IQ-tree. The phylogenetic tree was estimated using maximum likelihood with the Jones-Taylor-Thornton (JTT) model, and branch support estimates are based on 1000 bootstrap replicates. The legumes included are Arachis duranensis (V14167.a1), Arachis ipaënsis (K30076.a1), Cajanus cajan (Cc 1.0), Cicer arietinum (cicar.CDCFrontier.v1.0), Glycine max (Wm82.a2), Lotus japonicus (Lj3.0), Lupinus angustifolius (La1.0), Medicago truncatula (Mt4.0), Phaseolus vulgaris (V10), Trifolium pratense (Tp2.1), Vigna angularis (Va3.0), and Vigna radiata (Vr1.0). (PDF 1560 kb) [file 12870_2018_1467_MOESM3_ESM.pdf]

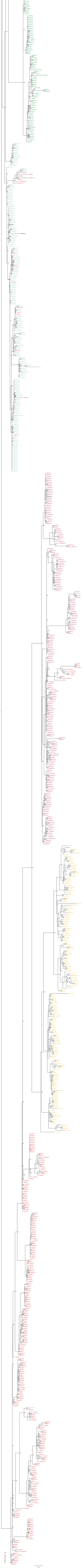

I-N
I-C
IIa
IIb
IIc
IId
IIe
III
0.3
